# Ovariohysterectomy in the Bitch

**DOI:** 10.1155/2010/542693

**Published:** 2010-03-07

**Authors:** Djemil Bencharif, Lamia Amirat, Annabelle Garand, Daniel Tainturier

**Affiliations:** Department of Reproductive Pathology, ONIRIS: Nantes-Atlantic National College of Veterinary Medicine, Food Science and Engineering, Site de la Chantrerie, B.P:40706, 44307 Nantes Cedex, France

## Abstract

Ovariohysterectomy is a surgical procedure widely employed in practice by vets. It is indicated in cases of pyometra, uterine tumours, or other pathologies. This procedure should only be undertaken if the bitch is in a fit state to withstand general anaesthesia. However, the procedure is contradicated if the bitch presents a generalised condition with hypothermia, dehydration, and mydriasis. Ovariohysterectomy is generally performed via the linea alba. Per-vaginal hysterectomy can also be performed in the event of uterine prolapse, if the latter cannot be reduced or if has been traumatised to such an extent that it cannot be replaced safely. Specific and nonspecific complictions can occur as hemorrhage, adherences, urinary incontinence, return to oestrus including repeat surgery. After an ovariectomy, bitches tend to put on weight, it is therefore important to inform the owner and to reduce the daily ration by 10%.

## 1. Introduction

Ovariohysterectomy in the bitch is a surgical procedure consisting of laparotomy with ablation of both ovaries and the uterus.

This procedure is indicated for the following [1].

- Uterine tumours.
- Serious uterine lesions, whether traumatic or infectious in origin; the most common cause being dystocia during parturition.
- Other pathologies that justify an ovariohysterectomy include metorrhagia, pyometria, glandular-cystic uterine hyperplasia with secondary infection leading to chronic metritis; the latter usually occurs postoestrus (“postoestrus metritis”) and is initially treated medically, as with acute postpartum metritis, surgery becomes a necessity once the disease becomes chronic and recurrent [2–5].

These alterations in the uterine mucosa are the result of ovarian hormonal imbalances.

Metritic pathologies have become increasingly common since the introduction and growing popularity of synthetic progesterone treatments such as medroxyprogesterone acetate, which are used to prevent or eliminate heats where the onset of metritis is common especially if they are used after the 3rd day of pro-oestrus [6].

This procedure should only be undertaken if the bitch is in a fit state to withstand general anaesthesia. She will reabsorb the toxins produced in the uterus, or lick any pus that accumulates at the lower commissure of the vulva, leading to gastroenteritis and hepatonephritis and subsequently diarrhoea, vomiting, and raised urea (normal value around 0.6 g/L), and creatinine (normal value around 10 mg/L).

However, if the ureamia is greater than 0.6 g/L, we advise the administration of Lespedeza capitata LESPEDEZIA N.D.v, 0.7–1 mL/kg morning and evening for 2 days via IM or SC injection, without exceeding 20 mL/injection/animal. The latter is a mild diuretic, hypoazotemic agent that acts via renal vasodilatation and stimulates the activity of the renal parenchyma. These injections should be combined with intravenous fluid therapy with isotonic NaCl solution at 0.9% and the urea levels checked 2 days later. Antibiotic prophylaxis with Cefalexin RILEXINE N.D.v, at a dose of 20 mg/kg every 12 hours during 3 days, is also advisable to prevent bacteraemia.

- Another indication is that of convenience, that is, sterilisation, as many owners complain of the manifestations of heat with vulvular discharge, as well as the problems associated with repeated matings.
- Finally, and with the owner's consent, ovariohysterectomy can be proposed as a radical alternative to medical abortion following an unwanted pregnancy, as it also involves definitive sterilisation [1].

However, there are certain contraindications to the procedure, such as if the bitch presents with a generalised condition with hypothermia, dehydration, and mydriasis. Similarly, animals presenting with hepatorenal insufficiency should not undergo general anaesthesia if the urea levels are greater than 0.6 g/L and the creatinine is greater than 10 mg/L, such animals are associated with poor peri- and postoperative survival. It is therefore essential to perform a complete, detailed preoperative clinical examination, with blood tests for serum biochemistry.

## 2. Materials and Method

### 2.1. Anatomy

**Figure 1:**
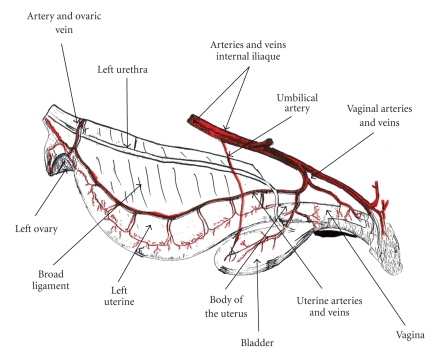
Arteries and veins of the genital apparatus of the bitch (Seen side left).

**Figure 2:**
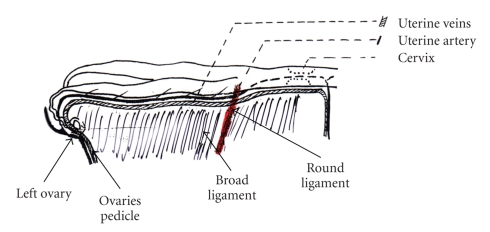
Uterine irrigation.

The genital apparatus of the bitch is primarily located in the abdominal cavity, with the exception of the vagina, which lies in the pelvis [6, 7] (Figures 1 and 2).

The neck of the uterus is relatively short, it measures 1-2 cm long, and lies a few centimetres in front of the anterior border of the pubis; it is followed by the body of the uterus, which measures 3–5 cm in length in the intrabdominal position, and which starts from the anterior straight of the pelvis then divides after a few centimetres into two divergent horns, which lie on the floor of the abdomen on either side of the linea alba, then travel back up towards the ovaries; the latter are situated in the costolumbar angle, one or two centimetres from the bisection and buried in a fatty ovarian sac, which opens medially [7, 8].

The uterus receives its blood supply from the right and left uterine arteries (Figures 1 and 2). The body of the uterus that lies closest to the oviduct is irrigated by the uterine branch of the ovarian artery, whilst the neck and remainder of the body are supplied by the uterine branch of the vaginal artery.

The uterine artery provides the majority of the organ's blood supply and serves no other organs; it originates from the internal iliac artery along with the umbilical artery.

### 2.2. Surgical Approaches

Easy access to the genital apparatus is gained via the linea alba; the incision starts at the umbilicus and ends 2 to 3 cm cranial to the anterior border of the pubis. This approach provides direct access to the uterine horns and facilitates prehension of the ovaries.

### 2.3. Surgical Technique

#### 2.3.1. Surgical Equipment

**(a) Preparation of the Animal**

Ensure that the bitch has been fasted since the previous day especially in the context of elective surgery: however, in an emergency situation, after induction of anaesthesia rapid intubation with a cuffed endotracheal tube should prevent aspiration of stomach contents due to gastric reflux.

In the event of pyometria or metritis, the bitch's organism has to eliminate the toxins produced during the infection, it is therefore essential for the success of the procedure to choose anaesthetic agents with minimal toxicity. Various protocols are available, these include: IV premedication with valium and morphine at 0.25 mg/kg and 0.1 mg/kg, respectively, followed by induction of anaesthesia with propofol at a dose of 5 mg/kg, the volume is increased slowly until the animal is sufficiently well anaesthetised to enable intubation. Isoflurane gas is then used to maintain anaesthesia; a flow rate of 2% is normally sufficient to maintain a good level of anaesthesia until the end of the procedure. To control perioperative pain, morphine can be administered at the same dose as for premedication, to a maximum of 10 injections/hour to avoid exceeding the threshold of toxicity.

Once anaesthetised, the bitch is positioned in dorsal recumbency with her front legs pulled forward and tied to the table, and the back legs tied back; the bitch is then put on a drip with previously warmed normal saline (0.9%) with glucose.

**(b) Preparation of the Surgeon**

The surgeon should wear a clean and sterile gown, scrub their hands thoroughly using surgical scrub solution, and wear sterile gloves.

**(c) Preparation of the Material**

In addition to the standard laparotomy kit, the surgeon requires the following instruments:

- 2 babcock forceps,
- 4 artery forceps,
- 4 doyen bowel clamps,
- resorbable multifilament suture material, VICRYL, dec. 3.

And finally material for the septic phase of the surgery: scalpel, mayo scissors, and resorbable VICRYL Dec 3.5 or 4 for closure of the abdominal wall.

#### 2.3.2. Surgery

The surgical zone should be carefully scrubbed using the same type of surgical scrub solution as used by the surgeon, and disinfected using alcohol and surgical antiseptic solution several times over.

**(a) Principal Phases**

**Laparotomy**

The skin is incised along the linea alba, that is, the sheath of the rectus abdominus, starting from the umbilicus and ending a few centimetres in front of the pubis.

Using a pair of scissors, the subcutaneous connective tissue, which may contain a substantial amount of fatty tissue, is bluntly dissected to visualise the linea alba.

Haemostasis is performed before opening the abdominal cavity. If simple swabbing proves insufficient, any bleeders should be ligated or twisted to obtain a very clean surgical field.

Using rat-tooth forceps, the linea alba is grasped in the middle and tented up before being incised with a pair of scissors. The peritoneum is then punctured using a cannula that is slid towards the umbilicus to enable incision of the linea alba without damaging the abdominal contents, with the cutting edge of the blade turned uppermost. The same procedure is then performed in the opposite direction towards the pubis.

If the uterine horns are voluminous they will be seen in the bottom of the surgical field following incision of the peritoneum; normal-sized horns will not be visible, for example, following recovery from postoestral metritis or during routine spaying.

To find the uterine horns easily, the operating table is tilted so that the animal's head is below its feet, to move the abdominal organs towards the diaphragm; this is known as the TRENDELENBURG position.

To locate the genital apparatus with ease, the bladder is retracted laterally; cranial to the bladder, the body of the uterus and bifurcation of the horns are easily locatable. One of the horns is then followed cranially up to the ovary, which is hidden in the fat-filled ovarian bursa. The ovary is not visible but can be felt through this ovarian bursa. It is a 1-2 cm long mass, which is exposed after incision of the bursa.

**Sectioning the Ovarian Pedicle and Broad Ligament**

– *Ovarian Pedicle*. The ovary is grasped and babcock forceps placed. The latter are handed to an assistant who holds the ovarian pedicle taught out of the abdomen to facilitate placement of a ligature as close as possible to the root of the pedicle to ensure haemostasis of the ovarian artery.
 The broad ligament is then punctured with a clamp to grasp the suture material and a ligature is placed in the ovarian pedicle as close as possible to the lumbar wall. Once this ligature has been placed, the ends of the threads are kept long so that the ovarian pedicle can be found with ease in the event of haemorrhage.
 A clamp is then placed between this ligature and the ovary, and the pedicle is sectioned between the two. The ovarian pedicle is held throughout this procedure with a clamp. The quality of the haemostasis is checked; the long ends of the suture material on the ovarian pedicle are then cut.
 In some cases, such as in the event of hypertrophy of the vascular bundle, it may be advisable to place two ligatures, one around the artery and one around the ovarian vein. Never hold the ligature itself with the clamp, as it might slip off the pedicle when being released back into the abdomen.
– *Broad Ligament*. If the broad ligament is seen to contain large vessels, they should be ligated prior to be being cut.
 However, if the vessels are invisible and buried under fat, the ligament can simply be torn in the middle above the uterine artery by exerting traction between two swabs with the fingers to tear it from front to back to the level of the cervix, and as close as possible to the lumbar wall. A point of resistance will be encountered within the round ligament; this corresponds to the vaginal process (which corresponds to the scrotum in the male) which explains the risk of inguinal herniation of the uterus in bitches following relaxation of the latter.
 Another technique for sectioning the broad ligament involves the placement of a row of overlapping mattress sutures along the length of the ligament before making the section with a scalpel or a pair of scissors.
 Once the ovarian pedicle has been sectioned, the second horn is located and the corresponding ovarian bursa grasped with Babcock forceps.
 The ovarian pedicle and broad ligament are sectioned as described previously.
 Finally, the two uterine horns are replaced back onto pelvis.

**Suturing the Anterior Portion of the Laparotomy Incision**

The prolapse of intestinal loops through the incision can cause significant heat and fluid loss, which can have very serious consequences, especially if the bitch is already suffering from deterioration in general status due to severe pyometria, for example. It is therefore advisable to suture the anterior portion of the laparotomy wound before continuing the surgery.

However, if the haemostasis of the ovarian pedicles or broad ligaments is a source of concern, the placement of a few forceps should suffice to provide temporary closure of the anterior portion of the laparotomy wound.

**Sectioning the Cervix [9]**

**Figure 3:**
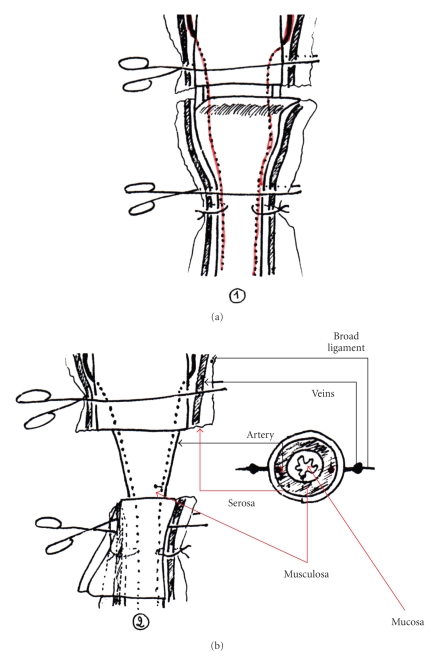
Section of the pathological cervix.

– *Ligating the Uterine Arteries and Veins (Figure 3)*. Once both uterine horns have been flipped back onto the pelvis, the uterine cervix is sectioned, following ligation of the uterine arteries and veins.
 The veins can be visualised passing on either side of the cervix. The arteries run under the veins in the musculosa of the cervix, which is why the haemostatic sutures should transfix the lateral walls of the cervix. However, if the uterine artery is perforated during ligation, a wider transfixion is needed, more caudal to the previous attempt.
– *Forcep Placement (Figure 3)*. Once both of the ligatures have been placed, the cervix is crushed at their level with an intestinal clamp. Another clamp is then placed just above the first and the contents of the uterus are pushed back towards the horns; two other clamps are placed in the same way above the 2nd clamp. The 2nd and 3rd clamps are removed, thus leaving a secretion-free zone.
– *Sectioning the Cervix (Figure 3)*. Once both intestinal clamps have been placed, the anterior section of the cervix is performed; the cervix may be normal or pathological.
   
*Normal Cervix*. The cervix is simply sectioned with a scalpel between the two clamps.
   
*Pathological Cervix*. For pathological cervixes, the serosa is dissected just caudal to the clamp that is placed on the uterus; the serosa is then retracted caudally.
 The musculosa is then sectioned cranial to the intestinal clamp placed on the cervix; if the clamps have been placed correctly, no fluid should leak from the cut ends.

**Dealing with the Stump (Figure 3)**

– *Small, Normal Cervix*. The stump is simply replaced in the abdominal cavity. It is however advisable to suture it or bury it in a fold of omentum.
– *Pathological Cervix*. The cut section of the musculosa, mucosa, is cauterised with an iodine-based solution, and then sutured in two phases:
   
*Septic Phase*. For the septic phase, a simple continuous suture is made in the musculosa with VICRYL N.D dec. 3.
   
*Aseptic Phase*. The needle is changed and either a buried simple continuous suture is made with the serosa (sero-serous continuous suture), or the stump is enfolded in one of the broad ligaments, which is fixated with a suture in the bursa. The ligament will weld itself to the stump. Finally, the stump can be invaginated by burying it in the vagina, then placing a ligature a few centimetres behind the original section. However, invagination is practically impossible to perform in small dogs due to the small size of their genital tract.
 These suture procedures eliminate the risk of peritoneal infection, since the pathological secretions drain into the vagina.

**Suturing the Abdominal Wall**

The sutured stump is returned to the abdominal cavity and the abdominal wall is closed using “X”-shaped interrupted sutures with VICRYL N.D. Dec.4.

If the subcutaneous connective tissue is very abundant, a simple continuous subcutaneous suture is performed using VICRYL N.D. Dec.3.

Finally, the skin is sutured using simple interrupted sutures or mattress sutures with non-resorbable filament such as MONOSIN N.D. Dec.3. The wound is then disinfected with antiseptic solution and protected with a few swabs and an adhesive dressing.

## 3. Results

### 3.1. Surgical Variation

Hysterectomy in the bitch via the linea alba is not very difficult. Nevertheless, it is sometimes necessary to perform the surgery via a vaginal approach rather than via the linea alba.

#### 3.1.1. Hysterectomy via the Vaginal Approach

Per-vaginal hysterectomy is performed in the event of uterine prolapse, if the latter cannot be reduced or if has been traumatised to such an extent that it cannot be replaced safely.

The elective site for amputation is between the cervix and urinary meatus, in which case there are two different possible techniques, either with an elastic ligature, or by suturing.

**(a) Elastic Ligature**

An elastic band is placed between the cervix and the urinary meatus, the exeresis is then performed and the stump sutured by joining the internal and external segments with a perforating simple continuous suture.

**(b) Suture**

Firstly, an intestinal clamp is placed between the cervix and the urinary meatus to crush the pedicle, then, either a transfixing suture or overlapping mattress suture is placed. Once the sutures have been placed, the vagina is excised.

The stump will be expelled within 15 days.

### 3.2. Postoperative Care

Firstly, advise perioperative oxygenation if the surgical shock is very great.

The animal is warmed, especially if the female was in poor condition prior to the procedure, she must be rolled in a blanket and placed in a heated kennel.

Intravenous fluid therapy is administered with isotonic saline along with an injection of Vitamin C and corticosteroids.

The bitch is then placed under antibiotic therapy for at least 5 days.

The sutures are removed after 10 days.

Any stagnant uterine secretions in the cervix and vagina will be eliminated in the days following and then cease completely.

### 3.3. Complications

These can be classified as general or specific.

#### 3.3.1. General

- Evisceration.
- Abdominal herniation.
- Suppuration from the cutaneous wound.
- Peritonitis.

#### 3.3.2. Specific

Haemorrhage: occurring during the intervention and continuing in the hours following. The latter represents one of the most common causes of the death of the animal. They can be situated at the level of the following.

**Figure 4:**
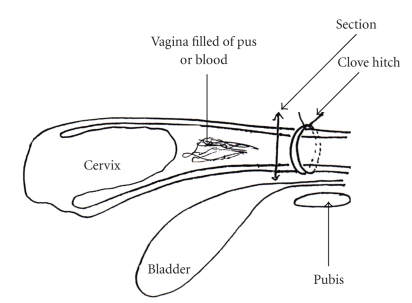
Complication: Pathological cervix amputation.

- *Ovarian pedicle*: this is why it is not advisable to operate during oestrus, where the uterine arteries and veins are hypertrophied.
- *Broad ligament*: always check that there is no significant haemorrhage after rupture.
- *Uterine cervix*: treatment involves a blood transfusion and repeat surgery to ligate the bleeding vessel.
- *Abscess*: These form especially at the level of the anterior straight of the pelvis and lumbar, when the cervix is not correctly treated, resulting in pain during defecation, vomiting, and finally an occlusive syndrome. The diagnosis is established via an exploratory laparotomy. Treatment is surgical, and may even necessitate nephrectomy or enterectomy.
- *Abdominal adherences*: these result from a localised peritonitis and cause an occlusive syndrome.
- *Urinary incontinence*: around 20% of spayed bitches are affected, especially large breeds.
- *Recurrence of metritis*: occurs in the month or years following the intervention, and a serous, purulent, or haemorrhagic vulvular discharge may occur. This complication occurs in bitches who have undergone hysterectomy alone, and which did not resolve the problem given that part of the genital apparatus remains in place: vagina, cervix, and occasionally a short section of uterus in front of the cervix, and when the oestrus cycle is abnormal, the uterine mucosa is not the only organ to suffer the consequences, the cervix and to a lesser extent the vagina also react. There are two possible solutions.
  - *Ablation of the cervix*: repeat surgery with placement of a clove hitch as far as possible from the cervix on the vagina. Then make a cut a few millimetres from the ligature. The stump is then disinfected with an iodine solution and sutured with a perforating simple continuous suture with VICRYL Dec. 3, and returned to the pelvic cavity. The disadvantage of this method is that the ovaries remain in place and the posterior portion of the vaginal mucosa may therefore continue to secrete abnormally for a few months, or even for up to a year later, which can result in the emission of drops of pus at the lower commissure of the vulva. This is why an ovariectomy is preferable and necessary (Figure 4).
  - *Return to oestrus*: the differential diagnosis should include cystitis, vaginitis, cervicitis, and inflammation or infection of the anal glands, which may be mistaken for signs of oestrus by the owners, which can be detected using a vaginal swab, and/or serum progesterone assay. If the bitch is confirmed as being in oestrus, there are two possible explanations: a residual fragment of the ovary, which was overlooked at the time of the ovariohysterectomy, or the secretion of oestrogens from the corticoadrenal glands due to hypophyseal dysfunction. Repeat surgery is therefore advisable, if the latter fails or if the surgeon is confident that no ovarian fragments have been overlooked then hormonal treatment can be started [6].
- Bitches who have undergone surgery for pyometria may present with thrombocytopenia: in the days following the procedure, the bitch presents with a marked tendency to haemorrhage. The mucosae are pale, the pulse weak, the bitch is hypothermic (36°C), and petechiae appear on the gingival and labial mucosae. Subcutaneous oozing is seen at the abdominal incision, and an angry red patch covering the entire caudo-ventral abdominal zone. Occasionally, a large quantity of blood clots is discharged from the vulva. Haematology reveals a significant reduction in circulating platelets, to the order of 3,500 rather than 300,000–450,000/mm^3^. The red blood cell count is also markedly reduced to around 120,000 instead of 6,000,000/mm^3^. The tendency to haemorrhage increases over time and the edges of the wound disunite within 3-4 days, the animal is very pale, comatose, and death follows.

## 4. Discussion

The current success rate is close to 95%, whilst several decades ago failures were to the order of 50%.

## 5. Conclusion

Ovariohysterectomy is the only effective treatment for pyometria and it is a radical treatment for postoestrus metritis when it recurs following failure of medical treatment. For the procedure to have the best chances of success, it is important to perform it on a bitch in good general condition.

It is also important to remember that after an ovariectomy bitches tend to put on weight, 1 kg for every 10 kg after 90 days; the metabolism of the bitch falls from 37 to 33 Kcal/day, it is therefore important to reduce the daily ration by 10%.
